# Associations between untraditional risk factors, pneumonia/lung cancer, and hospital fatality among hypertensive men in Guangzhou downtown

**DOI:** 10.1038/s41598-020-58207-z

**Published:** 2020-01-29

**Authors:** Yuechun Shen, Yuelin Chen, Zheng Huang, Junyao Huang, Xinchun Li, Zuojun Tian, Jun Li

**Affiliations:** 1 Departments of Cardiology, First Affiliated Hospital of Guangzhou Medical University, Guangzhou, Guangdong People’s Republic of China; 2 Department of Cardiology, Affiliated Zhongshan Hospital of Guangdong Medical University, Zhongshan, Guangdong People’s Republic of China; 3 Departments of Statistics, First Affiliated Hospital of Guangzhou Medical University, Guangzhou, Guangdong People’s Republic of China; 4 Departments of Radiology, First Affiliated Hospital of Guangzhou Medical University, Guangzhou, Guangdong People’s Republic of China; 5 Departments of Neurology, First Affiliated Hospital of Guangzhou Medical University, Guangzhou, Guangdong People’s Republic of China; 6 Departments of General Surgery, First Affiliated Hospital of Guangzhou Medical University, Guangzhou, Guangdong People’s Republic of China

**Keywords:** Cardiology, Health care, Medical research

## Abstract

Mortality of primary hypertension is high worldwide. Whether untraditional factors exist in modern life and affect the mortality is not well studied. The aim of the study was to evaluate the risk factors for fatality rate of hypertensive men in downtown area. A cross-sectional study was performed on hypertensive men, who were hospitalized into our hospital and lived in eligible urban areas. The characteristics of the patients and factors for the fatality were analyzed and of the risks or the contributors on the status were investigated. 14354 patients were identified. Mean age was 68.9 ± 12.4 year old (y) and dead ones was 75.9 ± 9.5 y. The overall hospitalized fatality was 5.9%, which was increased with age: fatality with 0.7%, 2.2%, 2.9%, 7.1%, 11.1% and 16.6% was for age group ≦ 49 y, 50–59 y, 60–69 y, 70–79 y, 80–89 y and ≧ 90 y respectively. The increased fatality was significantly positively correlated with the incidence of pneumonia, *P* < 0.05, r = 0.99. Pneumonia was prone to involve in men with older age and severer organ damage by hypertension. Similar to traditional risks such as coronary heart disease and stroke, pneumonia and lung cancer were also significantly associated with the fatality. Odds ratio (95% CI) for pneumonia and lung cancer were 6.18 (4.35–8.78) and 1.55 (1.14–2.11). The study provides evidence that pneumonia and lung cancer are highly associated with fatality of hypertensive men in downtown area, indicating that in order to reduce the fatality of hypertension, these lung diseases should be prevented and treated intensively in modern life.

## Introduction

Primary hypertension is one of the most common diseases worldwide; the prevalence reaches 60–80% in elderly^1^ and 26% in adult population^2,3^. It is also the most important risk for mortality, responsible for globally 13% of deaths^4^. Thus the both morbidity and mortality of hypertension are high incredibly in worldwide.

Traditional risk factors, such as coronary heart disease (CHD) and stroke, are implicated in the occurrence of death in patients with hypertension. However, the extent to which those risk factors correlated with mortality in such patients remain insufficiently assessed, highlighting a need for efforts toward finding untraditional risks in order to improve outcomes in this population.

With the social progress, economic development, population increase and human life spans lengthen ad so on, people’s lifestyle changes a lot. Modern life brings people fantastic world. However, the drawbacks exist, such as urban congestion and traffic pollution. One report^5^ showed that the proportions of men with hypertension in noisy and quiet areas were 23.6% and 17.5% respectively, The data of risk factors associated with mortality of hypertension in downtown community was deficient.

Pneumonia is among the most common illness^6^ affecting about 450 million people per year and occurring in every part of the world^6^. It is a main cause of death in all age groups resulting in 7% of the world’s total deaths per year^7,8^. Lung cancer is also common globally and accounts for the third highest prevalence in mortality^9^. Both pneumonia and lung cancer increase dramatically^10^.

Few data exist on whether pneumonia and lung cancer are associated with the mortality of individuals with hypertension. We have reported such issue among female hypertensive patients and found that pneumonia and lung cancer were associated with the fatality of certain age women with hypertension^11^. The object of this study was to assess the association between incidence of pneumonia/lung cancer and the fatality among male individuals with hypertension.

## Material and Methods

### Study design and data extraction

This was a cross-sectional study conducted at our hospital, the First Affiliated Hospital of Guangzhou Medical University, in Guangzhou of China. Male patients with primary hypertension were selected from those who were hospitalized into our hospital. The patients’ information forming variety data was derived from the database which officially recorded in information system in our hospital. It included patients’ age, gender, days of hospital stay, diagnoses, complications, comorbidities, therapeutic outcome, cause of death and place of residence, etc.

Ethical approval was obtained from ethics committee of our hospital, Medical Ethics Committee of the First Affiliated Hospital of Guangzhou Medical University. The chairperson of the committee waived the need for patient consent for this study since the study had no effect on the patients and their personal information was not identified in the study. The ethics committee reviewed and proved the study in accordance with International Conference of Harmonization, Good Clinical Practice principles and relevant national laws and regulations.

### Selection criteria

**Figure 1:**
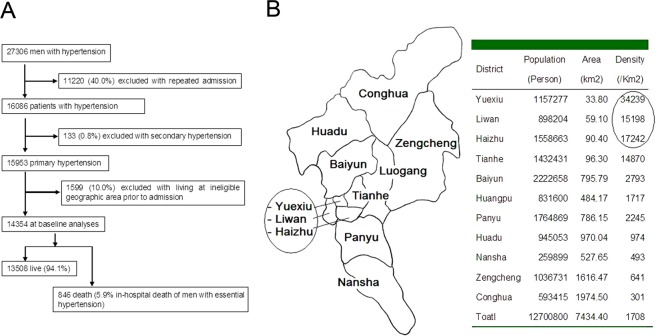
Data profile (**A**) and criteria for patients’ residence (**B**). (**A**) 14354 in-hospital hypertensive men are at baseline analyses after excluding ones who do not meet the criteria of the study. (**B**) A map of geographic districts in city Guangzhou and a list of population, area, and population density in the districts. The patients enrolled in current study are from three districts in circles, from where Yuexiu is our hospital located at. The map is generated by Microsoft (R) Paint, version 5.1 (2600, xpsp_sp3_afe.130704–0421: Service Pack 3).

The qualified hypertensive patients were consecutively selected from those who were hospitalized due to hypertension or with hypertension. Treaded uncontrolled hypertensives were included. In order to avoid potential sources of bias, patients who had any one of below conditions were excluded: repeatedly hospitalized (only last time was retained), with secondary hypertension, and lived in ineligible geographic areas prior to admission. How the study size was arrived was explained in flow diagram Fig. 1. The eligible geographic areas, in which Yuexiu is our hospital located at, and their population densities^12^ were shown in Fig. 1.

### Method of blood pressure (BP) measurement, diagnostic criteria of high BP and stage of high BP

The method of arterial BP measurement was manual cuff compression method with mercury column (Yuyue desktop vertical mercury sphygmomanometer, Jiangsu Yuyue Medical Equipment & Supply Co., Ltd., China). Subjects were empty their bladders and not allowed to smoke, drink tea or coffee for 30 minutes before BP was measured. They sat in a chair with a back, rest for at least 10 minutes to relax body and then BP was measured with sitting position. Both side upper arms were choices for the first time measurement; the side with higher BP value was taken and the side arm was choice thereafter for BP measurement. In general it was right side. Four limbs were measured when pulse pressure was larger than 20 mmHg to exclude secondary high BP. During the mercury drop of measurement, a value with clear first throb was systolic BP and with beat suddenly weak was diastolic BP.

Diagnostic criteria of high BP were: ①Patients had elevated systolic BP (SBP) ≥ 140 mm Hg, or diastolic BP (DBP) ≥ 90 mm Hg, or both BP ≥ 140/90mmg without antihypertensive medication for three times but not in one day. ②They had normal BP or controlled BP below 140/90 mmHg but taking antihypertensive agent/agents. Treaded uncontrolled hypertensives were included and secondary hypertensives were not included in the study. The criteria of stage I, II and III of hypertension were: Stage I - hypertension only; Stage II - hypertension with organ/organs damage but it’s/their function/functions was/were normal. Stage III - hypertension with organ/organs damage/damages as well as it’s/their function/fuctions was/were abnormal.

Organ/organs damage with it’s/their function/functions normal indicated organ/organs harmed by long-term load stress on small arteries resulting in insufficient blood supply due to high BP; it’s/their function/functions was/were normal by compensatory, in which the clinical manifestation/manifestations could be left ventricular hypertrophy, proteinuria or/and cerebral ischemia. Organ/organs damage with it’s/their function abnormal indicated the function/functions of organ/organs was/were decompensated at late stage, III stage, of high BP, such as cardiac failure, renal failure or/and cerebral stroke.

### Outcome observation

Primary outcome observed in the study was dead or alive. Secondary outcome observed was the risk factors, complications/comorbidities or diseases/disorders, which influenced the fatality. The associations of the factor/disease/disorder and the fatality of men with hypertension were analyzed. Diseases/disorders were categorized by ICD-10 codes (The International Statistical Classification of Diseases and Related Health Problems 10th Revision).

### Classification and definitions of diseases or disorders

In order to study associations of the risk factors and the fatality, the possible factors, complications/comorbidities or diseases/disorders, were classified as positive or negative. Their definitions were: CHD included all types of coronary heart disease. Diabetes did not include impaired glucose tolerance, because they were not the same. Cerebral infarctions were all conformed by brain computerized tomography (CT) or magnetic resonance (MR) examination.

Pneumonia indicated all types of pneumonia, including different location (lobar, bronchial and interstitial), and different cause (viruses, bacteria and/or fungi) etc. Tumor was malignant tumor including lung cancer etc; Lung cancers were all confirmed with histology; they included all types of cancers from the lung, also referred to metastatic lung cancer. Pneumonia or lung cancer might happen prior to or duration of hospital stay.

Hospital death indicated patients who were dead derived from either hypertension or other causes of death with hypertension. The fatality was all-purpose death with hypertension,

### Statistical analyses

Excel software was used to manage the data. SPSS software (Version 17; SPSS, Inc) was used to perform statistical analyses. Mean ± standard deviation was used to express continuous variables. Percentage or number was used to express categorical variables. Patients’ basic characteristics were described by Descriptive Statistics. Two continuous variables were compared by Mann-Whitney Test. Incidence of disease = (n/total population)X100%. Correlation analyses were performed with CORREL function to calculate Pearson’s correlation coefficient of r and with TTEST function to obtain statistical *P* value by Excel.

The associations of categorical variables and fatality were studied by Pearson Chi-square Test when count less than 5 with <20% cells expected; while they were studied by Continuity Correction when count less than 5 with ≧ 20% cells expected. Univariate Analyses were preformed first, from which only the significant variables (*P* < 0.05) were chosen and placed into Multivariate Analysis Model. Then Multivariate Logistic Backward Regression Model was applied to assess the associations between fatality and the factors, resulting in odds ratio (OR) and its 95% confidence interval (CI) calculated as the degree of fatality risk. *P* value of < 0.05 was considered statistically significant.

## Results

### Characteristics of selected patients

**Figure 2:**
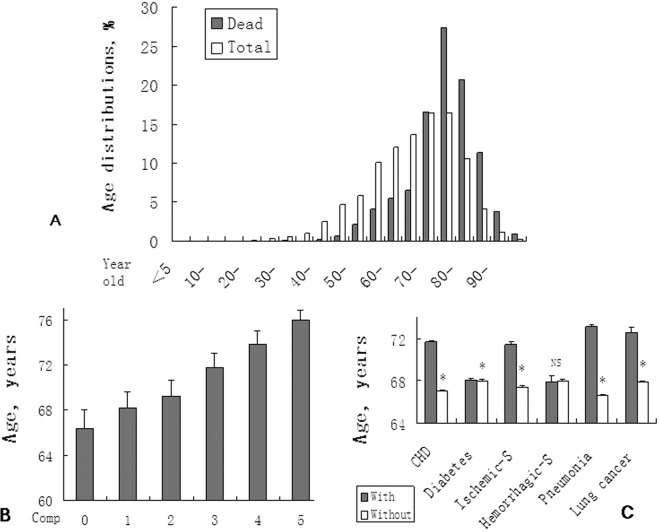
Age distribution of men with hypertension and dead ones (**A**) and relationship between the age and complications/comorbidities (**B**,**C**). (**A**) The figure supports hypertension is an aged related disease. (**B**) Numbers of Comp (complications/comorbidities) increase with age. *P < *0.000, r = 0.99. (**C**) Patients with Comp are significantly older than those without except for hemorrhagic stroke marked NS (not significant). *P < 0.05. CHD = coronary heat disease; -S = stroke.

A total of 27306 in-hospital men with primary hypertension were selected, Of which, 11220 (40.0%) was excluded with repeated admission; then 133 (0.8%) was excluded with secondary hypertension. The left patients were 15953. Of which, 1599 (10.0%) were excluded again with living at ineligible geographic area prior to admission. Finally, the sample size of 14354 was reached, Fig. 1. Mean age of the patients was 68.9 ± 12.4 year old (ys). The age distribution was gathered in 55–89 ys, in which, the percentage of each group, based on every 5 years, was >10%, Fig. 2.

### Characteristics of dead ones in selected patients

Among the patients in the study population above, overall fatality was 5.9%. Mean age of the death was 75.9 ± 9.5 ys. The age distribution was gathered at 70–89 ys, in which, the percentage of every group was >10%, Fig. 2A. The hospital stay and percentage of isolate hypertension (Patients who met the criteria for primary hypertension without any complication and comorbiditiy) from dead patients were shown in Table 1.

**Table 1:** Basic characteristics of hospitalized men with hypertension and dead ones. 5.9% is dead; from whom, only 0.6% patients present isolate hypertension.

Index	Men	Dead men
Sample (n, %)	14354, 100%	846, 5.9%
Mean age (years)	68.9 ± 12.4	75.9 ± 9.5
Hospital stay (days)	13.5 ± 15.5	19.1 ± 37.7
Age group (%)		
≦44	4.3	0.4
45–59	19.9	7.0
60–74	41.4	28.5
75–89	32.9	59.5
≧90	1.5	4.7
Isolate hypertension (n, %)	1778, 12.4%	5, 0.6%

### Leading complications and comorbidities

Patients accompanied with one or more complications/complications were as many as 87.6%, so patients with isolated hypertension were 12.4%. While, dead ones with isolated hypertension were only 0.6%, Table 1.

**Figure 3:**
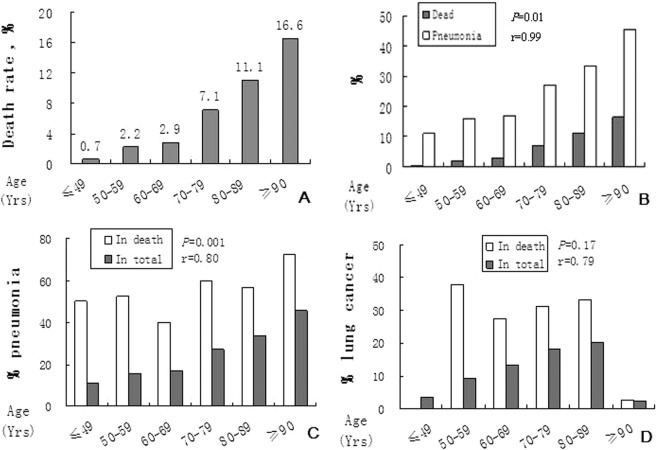
Positive correlation between incidence of pneumonia and the fatality of hypertension. (**A**), The fatality of men with hypertension increases with age. (**B**) Significant positive coefficient correlation (SP-CC) is found between incidence of pneumonia and the fatality. (**C**) SP-CC is also found in pneumonia between the patients and dead ones. (**D**) SP-CC is not found in lung cancer between the patients and dead ones.

Frequencies and types of complications/comorbidities were shown in Fig. 2 and Table 2. Except for traditional complications/comorbidities such as CHD and ischemic stroke, to our surprise, pneumonia and lung cancer were also among the leading complications/comorbidities list. For pneumonia, positive correlation was found between incidence of it and the fatality (Fig. 3).

**Table 2:** Leading complications/comorbidities in men with hypertension and dead ones. Except for traditional diseases such as coronary heart disease, pneumonia and lung cancer are also in the list.

Disorders	Men		Dead men	
	%	Age (Y)	%	Age (Y)
Coronary heart disease	20.7	70.3 ± 10.7	34.1	76.8 ± 8.7
Diabetes	22.9	67.1 ± 11.2	25.1	73.2 ± 10.6
Ischemic stroke	14.1	70.6 ± 10.7	19.4	76.4 ± 9.1
Hemorrhagic stroke	2.9	66.4 ± 13.3	7.5	74.7 ± 10.4
Pneumonia	16.7	72.3 ± 10.5	50.4	76.0 ± 9.1
Lung cancer	6.1	72.6 ± 8.9	17.4	74.6 ± 9.0
Acute diseases	17.1	72.0 ± 10.3	38.2	77.2 ± 8.2
Chronic diseases	27.4	71.6 ± 11.3	46.9	77.8 ± 7.7

### Factors influencing incidence of pneumonia and lung cancer

**Figure 4:**
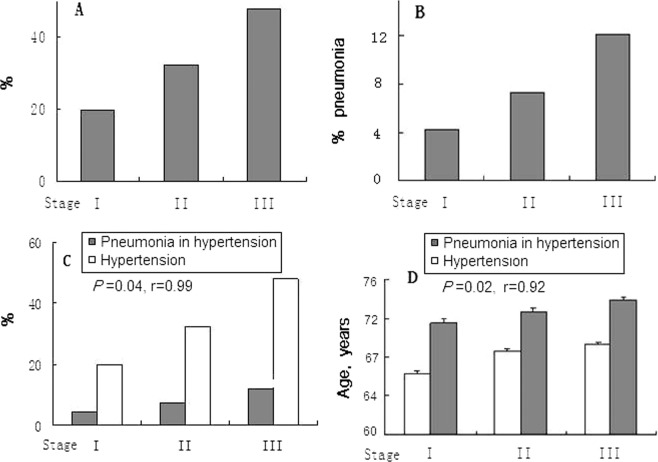
Pneumonia is susceptible in patients with higher stage of hypertension and older age. (**A**) Distribution of each stage of hypertension in the patients. (**B**) Distribution of pneumonia in each stage of hypertension. (**C**) The higher stage of hypertension the more pneumonia. (**D**) The higher stage of hypertension the more pneumonia and the older age.

In order to recognize what factors might affect incidence of pneumonia and lung cancer, we analyzed severity of the hypertension and age, and found positive correlation between incidence of pneumonia and stage of hypertension (Fig. 4), also between incidence of pneumonia and age (Fig. 4).

However, above correlations were not found for lung cancer. Percentage of stage I, II and III hypertension with lung cancer was 3.0%, 3.2% and 3.1%, *P* > 0.05. The mean age of men with lung cancer was 72.6 ± 8.9 (35–92) ys.

### Associations between traditional risks and the fatality

Thirty possible confounders which might affect the fatality were tested, Table 3. Firstly by univariate analysis, the positive factors, with *P* < 0.05, were applied into multivariate analyses. Secondly, by multivariate analyses, older age, hemorrhagic disorders, acute diseases, chronic diseases and tumor, as broad categories, were found significantly associated with the fatality. In addition, CHD, diabetes, cerebral infarction and acute cerebral hemorrhage were also associated with the fatality. all *P* < 0.05. It’s reasonable that they were widely accepted as traditional risks for fatality of hypertension.

**Table 3:** Unadjusted odds ratios (OR) and 95% confidence intervals (CI) by univariate and adjusted OR and CI by multivariate analyses for risk factors associated with the fatality of hypertension.

Analyses	Men with hypertension			
	Univariate		Multivariate	
Variables (Risk factors)	*P*	Unadjusted OR (95% CI)	*P*	Adjusted OR (95% CI)
**Age** (years old)				
≦59	0.000	0.24 (0.18–0.31)		
60–74	0.000	0.55 (0.47–0.64)	0.012	1.46 (1.09–1.96)
75–89	0.000	3.23 (2.80–3.72)	0.000	3.05 (2.3–4.06)
≧90	0.016	3.65 (2.57–5.17)	0.000	5.16 (3.26–8.17)
**Cardiovascular diseases**				
Coronary heart disease	0.000	2.09 (1.80–2.42)	0.000	2.12 (1.79–2.50)
**Metabolic diseases**	>0.05			
Dyslipidemia	>0.05			
Diabetes	0.002	1.14 (1.00-0.33)	>0.05	
Obesity	>0.05			
**Cerebrovascular diseases**	0.000	1.44 (1.24–1.68)	>0.05	
Cerebral infarction	0.000	1.48 (1.24–1.77)	0.000	1.59 (1.30–1.94)
Vertebrobasilar insufficiency	>0.05			
Cerebral hemorrhage	0.000	2.95 (2.23–3.89)	>0.05	
Acute cerebral hemorrhage	0.000	3.52 (2.57–4.83)	0.000	2.91 (1.47–5.77)
**Hemorrhagic disorders**	0.000	3.68 (3.01–4.51)	0.000	3.13 (1.80–5.47)
Other hemorrhagic disorders	>0.05			
Gastrointestinal hemorrhage	0.000	4.79 (3.60–6.37)	>0.05	
**Acute diseases**	0.000	3.31 (2.86–3.83)	0.000	1.82 (1.49–2.23)
**Chronic diseases**	0.000	2.49 (2.17–2.87)	0.000	1.54 (1.25–1.90)
**Inflammatory or infectious diseases**	0.000	4.31 (3.70–5.12)	>0.05	
Tuberculosis	>0.05			
Pulmonary infection	0.000	6.20 (5.34–7.20)	>0.05	
Pneumonia	0.000	7.33 (6.33–8.48)	0.000	6.18 (4.35–8.78)
Tracheobronchitis	>0.05			
COPD	0.000	2.05 (1.70–2.48)	>0.05	
**Gastrointestinal diseases**	>0.05			
Biliary gallbladder disease	>0.05			
Upper gastrointestinal disease	0.000	1.63 (1.28–2.10)	>0.05	
**Tumor**	0.000	2.38 (2.04–2.80)	0.000	3.29 (2.69–4.02)
Lung cancer	0.000	4.47 (3.49–5.72)	0.006	1.55 (1.14–2.11)

^1^Multivariate represents multivariate logistic backward regression analysis, adjusting for other factors which are significant (*P* < 0.05) in univariate analysis.

^2^Age is divided into four sub-groups. In multivariate analysis, last three sub-groups are compared with the first sub-group separately and their fatality risk increase 1.46, 3.05 and 5.16 times.

^3^ Except for traditional risks, pneumonia and lung cancer are also significantly associated with the fatality of men with hypertension.

^4^ OR = odds ratio; CI = confidence interval; COPD = chronic obstructive pulmonary disease.

### Associations between untraditional risks, pneumonia/lung cancer, and the fatality

**Figure 5:**
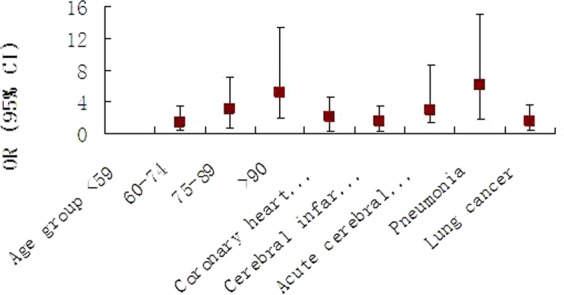
Associations of risk factors with the fatality of hypertension. As age increases, the risk of the fatality increases. Similar to traditional risks such as coronary heart disease, pneumonia and lung cancer are also risks for the fatality of hypertension.

Except for above risks, pneumonia and lung cancer were also found significantly associated with the fatality, both *P* < 0.05. Furthermore, pneumonia presented odds ratio number the highest, OR = 6.18, 95% CI = 4.35–8.78, indicating it was the most serious factor associated with the fatality. Lung cancer presented OR also >1, = 1.55, indicating the factor was a risk factor, 95% CI = 1.14–2.11, Table 3 and Fig. 5.

It is important to know how many patients were hospitalized with pneumonia/lung cancer and died with it and how these related to the study population. The answers were: 8.0%/3.9% patients were hospitalized with pneumonia/lung cancer, 24.8%/9.6%% patients were died with it (pneumonia/lung cancer). Of the current study population, 16.7%/6.1% patients were hospitalized with pneumonia/lung cancer; 50.4%/17.4% patients were died with it (pneumonia/lung cancer). The former was less than the latter: hospitalized total patients with pneumonia/lung vs. hospitalized hypertensive patients with pneumonia/lung = 8.0% vs. 16.7% /3.9% vs. 6.1%, *P* = 0.06/0.48; for dead ones = 24.8% vs. 50.4%/ 9.6% vs. %17.4%, *P* = 0.0002/0.11. Of the four *P* values, hospitalized hypertensive dead patients with pneumonia were significantly more than hospitalized total patients with pneumonia, *P* < 0.05 = 0.0002; supporting pneumonia is significantly affect the fatality of hypertension.

## Discussion

The mortality of hypertension is very high^4^ and associated with some factors. The most studied and recognized factors are cardio-cerebral-vascular and metabolism related risk factors, such as CHD and stroke, arterial ischemic stroke, diabetes^13^, serum uric acid^14^ and coronary artery calcium^15,16^. Untraditional risk factors are assessed insufficiently although some of them are also reported including chronic obstructive pulmonary disease (COPD)^17^, physical activity^18^, geographic concentrations of medical doctors^18^, sex and ethnicity^19^. Whether pneumonia and lung cancer affect the mortality of hypertensive patients is uncertain. We have reported^20^ they were significantly affect the mortality of certain age women with hypertension.

In the study, we found the most severe risk factor associated with the fatality of men with hypertension was pneumonia. In literature we could not found this information directly, but below evidences indirectly supported our conclusion: Pneumonia is very common ① in all parts of the world^7,8^; ② in causes of death accounting for 7% of the total death globally yearly, and 10–25% of the hospital deaths who were particularly most in older people and in patients with comorbidities^21,22^; ③ in hospitalized patients with cardiovascular diseases and with a increased trend to poor outcomes^23,24^.

Thus, pneumonia is a disease very common and results in high fatality. Coincidentally, hypertension shares these characteristics as well. Therefore, patients should have worse outcomes when they suffer from both hypertension and pneumonia. We also found positive correlations between incidence of pneumonia and age, and between incidence of pneumonia and stage of hypertension. These could explain why and what types of patients (with older age and severe condition of organ damage by hypertension) were prone to involve in pneumonia.

Except for pneumonia, we found another significant risk affect the fatality - lung cancer. Similar to hypertension, lung cancer is also a disease of the elderly; most of the patients with lung cancer (>65%) are old (>65 years old)^25^. Our results were consistent with this that 75.4% of patients with lung cancer were ≧65. In addition, lung cancer is the most common among cancers for men in Asia^26^ and the world (1.1 million, 16.5% of all)^27^. Such high incidence of lung cancer makes it important to reduce its harmfulness.

Hypertension is reported related to increased risk modestly for cancer incidence and mortality^28^. Medication for lung cancer could make blood pressure higher or cause the outcomes worse, such as Bevacizumab^29^ and Regorafenib. They may result hypertension, blisters on hand or/and foot skin, and diarrhea. These side effects might be manageable by reducing dosage or stopping the medicines^30^. Therefore, doctors should beware of the side effects of the medication, prevent and treat them in time.

Causal relationships of hypertension to pneumonia, and between pneumonia and lung cancer may exist. These include: (1) hypertension increases cardiovascular risks such as stroke, which is related with pneumonia: approximately one third of patients with hypertension occurs pneumonia after acute stroke^31^. (2) hypertension induces vascular endothelial dysfunction with oxidative stress and reactive oxygen species, which are key signaling molecules playing an significant role in the development of inflammation including pneumonia^32^. (3) hypertension causes kidney failure and heart failure etc. leading to low immunity and respiratory tract infection. Besides, patients with lung cancer increase chances of developing pneumonia; while, patients with history of lung diseases present increased risk of having lung cancer^33^. All in all, inner-link among hypertension, pneumonia and lung cancer may exist. This may explain why pneumonia and lung cancer are risks significantly affect the fatality of hypertension.

In addition, the reason for high incidence of pneumonia and lung cancer with hypertension in the study should be related with the adverse environment condition of the patients, who were all from the top three most crowd districts in city Guangzhou. These districts have high population density; this city is arranged at the third place for large city in China, and is an important trading port and national transportation center, causing heavy air pollution. Hence, air pollution and carcinogenic material increased etc, should contribute to the more incidence of pneumonia and lung cancer. For history of smoking habits as risk factors, we should consider but didn’t include due to a large number of patients analyzed in the study. The database we used didn’t include this parameter and it’s difficult to collect this data in a short period of time from tens of thousands of people. We may study this in the future.

How to reduce the mortality and fatality of hypertension? Some organizations for the deal with hypertension emphasized to lower blood pressure, but overlook the complications/comorbidities of hypertension. Our observation indicated that for hypertensive management, taking care of complications and comorbidities is extremely important to decrease mortality/fatality, not only traditional risks such as coronary heart disease and stroke, but also lung diseases like pneumonia and lung cancer.

## Conclusions

In conclusion, we studied hospitalized fatality of hypertension and effecting factors among a large number of patients in urban China. Our findings provided evidence on fatality and factors associated with fatality; especially the untraditional factors. This study suggests that in order to reduce the mortality/fatality, men with hypertension need intensively management for prevention, diagnosis and treatment of pneumonia and lung cancer.
